# Exploring Novel Foods: Consumer Willingness and Acceptance of Edible Insects as a Sustainable Alternative Protein Source in Saudi Arabia

**DOI:** 10.3390/foods14152590

**Published:** 2025-07-24

**Authors:** Samar Refat Alabdulmohsen, Hala Hazam Al-Otaibi

**Affiliations:** Department of Food and Nutrition Science, College of Agricultural and Food Science, King Faisal University, Al-Ahsa 31982, Saudi Arabia; 223002340@student.kfu.edu.sa

**Keywords:** edible insects, willingness, acceptance, consumer, alternative protein, food neophobia, Saudi Arabia

## Abstract

Edible insects are emerging as a sustainable and eco-friendly alternative protein source, addressing global challenges in food security and environmental sustainability. This study investigates the willingness and acceptance of edible insects as sustainable alternative protein sources among Saudi participants, focusing on sociodemographic and attitudinal factors. A cross-sectional survey involving 2208 participants revealed that males and younger individuals (18–34 years) exhibited greater willingness to consume EIs, driven by environmental concerns, health benefits, and positive beliefs. Women and older participants showed higher levels of food neophobia, disgust, and uncertainty. Sociocultural barriers such as fear, a lack of familiarity, and perceptions of edible insects as unclean or forbidden were significant obstacles to acceptance and willingness. Interestingly, the education level did not significantly influence willingness, suggesting that cultural and religious norms may overshadow its impact. These findings underscore the need for targeted campaigns emphasizing the health, environmental, and sustainability benefits of edible insects, alongside culturally sensitive approaches to overcome psychological and social barriers. This research provides valuable insights to guide strategies for integrating EIs into Saudi diets as a step toward enhanced food security and environmental sustainability.

## 1. Introduction

Edible insects (EIs) have been consumed in various cultures and religions worldwide since ancient times and have been a component of human nutrition. In the 1970s, Meyer-Rochow suggested that the Food and Agriculture Organization (FAO) and World Health Organization (WHO) should explore EIs as rich sources of protein that can be used to address global protein shortages and food security [1]. Later, in 2013, FAO published the book “Edible insects: future prospects for food and feed security” [2] and discussed the importance of EIs as nutritional foods for humans owing to its environmentally friendly, safe, and sustainable characteristics, and suggested its legislation.

In September 2015, the United Nations (UN) adopted the 2030 Agenda for Sustainable Development Goals (SDGs), which included 17 goals to address global challenges such as food security, food sustainability, and climate change, with a focus on preserving the planet’s resources. The shift towards a circular economy is essential to achieving these goals, given the close relationship between the planet’s resources and protein supplies [3]. UN reports indicate that the world’s population will continue to increase, reaching 8.4–8.7 billion by 2030 and between 9 and 10 billion by 2050 [4]. The FAO anticipates the food demand to increase to 60% by 2050 [5]. The consumption of EIs constitutes a potential strategy for addressing food security and sustainability challenges, representing an innovative and ancient approach to the global environmental challenges. Currently, EIs are part of the human diet with almost 2000 species consumed, either directly (raw or cooked) or indirectly (powder added to biscuits and bread), in many parts of the world [6,7]. Although commonly consumed in Asia, Africa, and South America, they are relatively unexplored in Western societies, such as Europe and North America [8].

EIs are novel, highly nutritious, and excellent sources of protein, providing 40–75 g of protein per kg; moreover, they are rich in essential amino acids [9], fats (13–33%), and fiber, with small quantities of saturated fatty acids [10]. In addition, EIs have high proportions of minerals such as iron, calcium, zinc, magnesium, copper, and phosphorus as well as vitamins such as biotin, riboflavin, pantothenic acid, and folic acid [11]. Furthermore, EIs contain biologically active compounds that can strengthen the immune system function and reduce health risks [12].

EIs are environmentally friendly and contribute to pollution reduction due to the recycling of organic waste and the significant reduction in greenhouse gas emissions compared to livestock farming [13]. They are highly efficient in converting food into body mass, as they can convert 2 kg of feed into 1 kg of body weight, compared to cows that require approximately 8 kg of feed to gain 1 kg weight [14]. In addition, insects require less water and feed to produce protein and help in reducing the use of pesticides, thereby reducing environmental pollution and pesticide residues in food [13].

However, several factors limit consumer acceptance of EIs as sources of protein, such as psychological factors owing to the lack of familiarity that results in disgust and neophobia. A strong relationship has been reported between high levels of food phobia and abstention from consuming insects [15,16,17]. Food neophobia is defined as the fear of eating unfamiliar foods and is associated with cultural and social norms [18]. Disgust toward eating insects is related to the anticipation of a negative consumption experience and an expected sense of discomfort, primarily owing to their appearance often being perceived as dirty and fears of contamination and disease [19]. Willingness and positive attitudes play a major role in shaping our dietary habits as they influence our beliefs and experiences [17]. Furthermore, fostering positive attitudes towards EIs and recognizing sustainable, safe protein sources can support their incorporation into the human diet, contributing to the advancement of sustainable practices [20].

Sociodemographic factors impacting consumer acceptance include sex, with men having a higher positive attitude level than women. This observation may be owing to the higher neophobia among women in various cultures [21]. Considering age, younger participants are more open towards EIs consumption, particularly those with a higher level of education and knowledge about the nutritional advantages of EIs consumption. In addition, individuals with a higher income are more receptive to novel experiences [22,23].

Cultural acceptance varies significantly across regions, influenced by factors such as tradition, awareness, and exposure to insect-based foods. China, a country with a long history of consuming insects, highlight their acceptance as part of traditional diets in certain regions. Liu et al. [23] indicate that urban Chinese participants are gradually becoming more receptive to EIs, particularly when presented as processed products, such as powders. The factors influencing acceptance include perceived knowledge about nutritional benefits, safety, having a higher income, age, and a larger household size.

In Malaysia, the practice of eating EIs is a notable part of the food culture, particularly in inland communities. Lim et al. [24] identified key factors influencing the consumer acceptance of edible insects, including safety and texture. Their research also revealed that male participants showed a greater acceptance of insects as food compared to female participants. Furthermore, non-Muslim individuals were more open to consuming insects than Muslims, who tend to exercise greater caution with food choices, especially when encountering novel or unfamiliar items.

Conversely, in European countries, where EIs consumption is less common, acceptance is often hindered by neophobia and cultural perceptions. Research by Megido and colleagues [20] in Belgium showed that the acceptance of edible insects was higher when the insects were processed into unrecognizable forms, such as insect-based burgers or powders. However, it is important to note that their study focused on individuals identified as ‘future insect consumers’ participants who already expressed some openness to insect consumption. Therefore, the findings may not reflect the general population’s attitudes, and broader consumer acceptability, especially among those unfamiliar or hesitant, remains a significant challenge.

Similar findings were reported in Germany, where Hartmann and Siegrist [21] observed that awareness campaigns emphasizing sustainability and nutritional benefits positively influence attitudes toward EIs foods.

Saudi Arabia (SA) is characterized by rapid economic development, continuous population growth, and urbanization. SA requires an economy aligned with the SDGs and Saudi Vision 2030, which aims to create a thriving economy, a vibrant society, and an ambitious nation. Some of the Saudi Vision 2030 goals align with goal 2: zero hunger; goal 12: responsible consumption and production; and goal 13: climate action, emphasizing a balance between economic growth and sustainable development with a focus on environmental and climate changes.

To enhance food security, especially with a limited area, water, and arable land for agriculture, SA faces challenges in providing sufficient food for its population, particularly protein. Similar to many other countries, SA has been exploring sustainable food resources that will provide food security without environmental stress. SA has eight indigenous insect orders, high in protein and suitable for consumption, including Orthoptera, Diptera, and Coleoptera [25]. Historically, locusts are commonly consumed in all Saudi Provinces and are recognized as a halal food that can be eaten as a traditional protein source [26]. With one of the fastest growing economies in the world, SA has had a pronounced transition from traditional to modernized diets and lifestyles. Locust consumption is increasingly less favored than traditionally practiced.

To the best of our knowledge, no previous studies have explored Saudi consumer willingness to accept of EIs as alternative protein sources. This study aims to evaluate the impact of sociodemographic and attitudinal factors on the consumer acceptance of EIs. Additionally, it aims to examine the potential predictors influencing the willingness to accept EIs as alternative protein sources among Saudi participants.

## 2. Materials and Methods

### 2.1. Study Design and Participants

This cross-sectional study was conducted in SA between October 2024 and January 2025. Participants were recruited through snowball sampling method on popular social media platforms (WhatsApp, X platform, and Telegram) and electronic questionnaires were administered using a Google Form link. Word-of-mouth recruitment was used to encourage individuals to participate in the study, and they were requested to share the survey within their social networks. To increase the diversity of the study sample, the snowball sampling approach was initiated with diverse initial participants from all five main administrative regions of Saudi Arabia (Eastern, Central, Western, Northern, and Southern). Efforts were made to recruit individuals from varied demographic backgrounds—including age, sex, education, and employment—across different geographic locations. This was facilitated through targeted distribution on widely used social media platforms and collaboration with initial participants from multiple cities and provinces, thereby ensuring broad national coverage. Before filling out the questionnaire, a brief sentence was included explaining the objectives, instructions for completion, and a consent process, emphasizing the confidentiality and anonymity of their responses. The inclusion criteria required participants to be (1) Saudi nationals, (2) aged 18 years or older, and (3) residing in Saudi Arabia at the time of this study. Responses that did not meet these criteria or were incomplete were excluded during the screening process. According to the data from the 2020 General Authority for Statistics, there are 23,426,485 million citizens aged ≥18 years in SA. The sample size was calculated based on an expected correlation coefficient of 0.15, a strength of 80%, and an alpha level of 0.05; thereby, a minimum of 385 participants were required. The initial sample size reached 2229 participants. After screening according to the eligibility criteria, the final number became 2208 participants.

### 2.2. Study Questionnaire

The questionnaire items used in this study were adapted from previous studies, prepared in English, and subsequently translated to Arabic by bilingual professionals using a forward–backward translation process. A pre-test was conducted among 20 Saudi adults to ensure clarity, coverage of all study aspects, ease of use, and to establish the feasibility and validity of the questionnaire; these results were excluded from the final data analysis.

The questionnaire comprised two main sections. The first included six questions about sex, age, education, employment status, income, and region of residence, and one question on previous experience with EIs consumption: “Have you eaten insect-based foods? with “yes” or “no” response” [17]. The second section included acceptance and eight attitudinal factors: one question addressing familiarity—“Have you ever met or heard of someone consuming edible insects, such as locusts”—and one question addressing social effects of EIs as alternative protein sources—“I think I can taste edible insects if I see everyone eating them” with “yes” or “no” response [27,28]. Barriers were assessed by five questions asking participants to identify the most common reason for avoiding EIs as an alternative source of sustainable protein, with “yes” or “no” response [29,30].

Acceptance was measured by assessing participants’ willingness to consume EIs, which included four items: “If served well-cooked edible insects, as pasta, I would try them”; “I would like to eat flour fortified with proteins that come from insects”; “I might eat insects in the future, they taste great”; and “I am open to buying food with added dyes or insect-based protein.”. Responses were measured on a five-point Likert scale ranging from 1 (“strongly disagree”) to 5 (“strongly agree”) [21,30]. Participants’ willingness scores have a possible range of between 4 and 20, with willingness ranges obtained using the willingness mean scores from this study plus or minus standard deviation. The mean and standard deviation for the present study was 6.98 ± 3.35 and as such, participants were classified as unwilling to consume EIs as alternative protein sources with a total score of ≤4, as uncertain with scores between 5 and 11, or as willing to consume EIs as alternative protein sources with scores of ≥12.

The Food Neophobia Scale (FNS) questionnaire is a validated tool widely recognized as the standard method for measuring levels of food neophobia, developed by Pliner and Hobden [31]. It has been employed in other studies [31,32,33] and adapted for the participants in the current study: “Ethnic food looks too weird to eat,” and “I don’t trust new foods” [34]. These 10 items were evaluated on a seven-point Likert scale (1 = “I strongly disagree” and 7 = “I strongly agree”) [31,32].

Environmental concerns were evaluated using two items: “I try to reduce my impact on the environment through the choice of food, such as EIs,” and “Insects efficiently convert organic matter into protein.” Participants rated their agreement on a scale ranging from 1 (“strongly disagree”) to 5 (“strongly agree”) [17].

To evaluate perceptions of the health benefits of edible insects (EIs) as protein sources, three items were included: “Consuming insects as natural snacks provides a rich source of essential amino acids”; “Fortifying bread and pasta with insect-derived proteins boosts their nutritional value and supports health”; and “Eating protein sourced from insects is good for health.” Responses were recorded on a five-point Likert scale ranging from 1 (“strongly disagree”) to 5 (“strongly agree”) [27].

Belief was assessed with three negative items, such as “Some people would have allergic reactions to eating insects”; “Eating insects would expose me to harmful chemicals and insecticides”; and “Insects carry harmful microbes” [30]. Responses were measured on a five-point Likert scale, with 5 indicating “strongly disagree” and 1 indicating “strongly agree” [30].

Weighted means were computed for the seven-point and five-point Likert scale scores by assigning varying weights to each response option, enabling more detailed interpretations of survey data, particularly when distinguishing between levels of agreement or disagreement is essential [34,35]. The internal consistency for the scales measured using Cronbach’s alpha coefficients ranged from 0.904–0.908.

### 2.3. Statistical Analysis

Data were coded using Excel Spreadsheet, then analyzed using IBM SPSS Statistics for Windows, version 29 (IBM Corp., Armonk, NY, USA). The normality of the data was assessed. Descriptive analyses were used to calculate means, standard deviations, percentages, and frequencies. Advanced statistical analysis tests were performed, including comparison tests such as chi-square for categorical variables, for more than two independent groups, one-way ANOVA was applied, and independent *t*-test was used for two independent groups. Multiple regression was used to examine the potential predictors influencing willingness to consume EIs as alternative protein sources among Saudi participants. Statistical significance was set at *p* < 0.05 willingness to accept.

## 3. Results

The total sample comprised 2208 participants (Table 1). Most of the participants were younger in age (44.57%), had a higher education, were employed, and earned more than 10,000 SR per month. The geographical distribution showed a relatively balanced spread, with the highest proportion of participants from the central region (32.9%) and the lowest from the northern region (5.9%).

Table 2 presents the associations between the socio-demographic characteristics, experience, and familiarity of participants with EIs. The results showed a statistically significant difference between the sexes, with men (24.1%) and the younger age group (14.7%) having more experience of eating EIs than women (7.8%). Regarding familiarity, we asked the participants if they knew or had met someone who had eaten EIs such as locusts; most had known or met someone who had consumed EIs, with a non-significant difference between men and women and the level of education. However, there was a statistically significant difference between the age groups (*p* = 0.000).

For the social effects, participants were asked whether they would try EIs if they saw others consuming them (Table 2). There were statistically significant differences between the sexes (*p* = 0.000) and age groups (*p* = 0.000). The first barrier to eating EIs as a sustainable protein source was disgust and feeling like it is unacceptable (51.6%). More women (55.6%) significantly expressed disgust and considered EIs as unacceptable (*p* = 0.000), with the highest proportion within the 26–40 age group, where 57.6% shared this view (*p* = 0.015) (Table 2). The second most common barrier reported by 17.6% of respondents was that there are better and healthier food options than EIs as sustainable protein sources. Men (23.2%) reported this barrier more than women (15%) (*p* = 0.000).

Health risks were the third most common barrier (12.8%), with more men (18.5%) considering that consuming EIs may be unsafe or carry unknown health risks compared to women (9.8%), with a statistically significant difference (*p* = 0.000). The fear of EIs was significantly more common among women (15.9%) than men (5.7%) and the younger age group (13.7%) (*p* = 0.000, *p* = 0.002, respectively). EIs were considered forbidden (not Halal), and hence, should not be consumed, so this was also reported as a barrier by 5.4% of respondents. This barrier was most prevalent among individuals aged ≥ 55 years (*p* = 0.006) and among those with a secondary school education or lower (*p* = 0.004).

**Table 2:** Associations between socio-demographic factors, experience, and familiarity, social effects and barriers of participants (*n* = 2208).

^a^ Experience				
Have you eaten insect-based foods?				
Sociodemographic variables	Category	No (%)	Yes (%)	*p*-value
Sex	Male	575 (75.9)	183 (24.1)	0.000 ***
	Female	1337 (92.2)	113 (7.8)	
Age (years)	18–34	1460 (85.3)	251 (14.7)	0.002 *
	35–54	335 (92.3)	28 (7.7)	
	≥55	117 (87.3)	17 (12.7)	
Level of education	≤Secondary school	233 (84.4)	43 (15.6)	0.257
	≥University	1679 (86.9%)	253 (13.1)	
^a^ Familiarity				
Have you ever met or heard of someone consuming edible insects, such as locusts?				
Sociodemographic variables	Category	No (%)	Yes (%)	*p*-Value
Sex	Male	172 (22.7)	586 (77.3)	0.293
	Female	301 (20.8)	1149 (79.2)	
Age (years)	18–34	327 (19.1)	1384 (80.9)	0.000 ***
	35–54	97 (26.7)	266 (73.3)	
	>55	49 (36.6)	85 (63.4)	
Level of education	≤Secondary school	58 (21.0)	218 (79.0)	0.860
	≥University	415 (21.5)	1517 (78.5)	
^a^ Social effects				
I think I can taste edible insects if I see everyone eating them				
Sociodemographic variables	Category	No (%)	Yes (%)	*p*-value
Sex	Male	506 (66.8)	252 (33.2)	0.000 ***
	Female	1228 (84.7)	222 (15.3)	
Age (years)	18–34	649 (76.2)	235 (23.8)	0.000 ***
	35–54	656 (87.6)	93 (12.4)	
	≥55	400 (84.3)	75 (15.7)	
Level of education	≤Secondary school	205 (74.3)	71 (25.7)	0.066
	≥University	1529 (79.1)	403 (20.9)	
^a^ Barriers				
Unsafe or may carry unknown health risks (*n* = 284; 12.8%)				
Sociodemographic variables	Category	*N*	(%)	*p*-Value
Sex	Male	140	18.5	0.000 ***
	Female	142	9.8	
Age (years)	18–34	121	12.5	0.784
	35–54	101	13.8	
	≥55	62	13.4	
Level of education	≤Secondary school	40	14.5	0.36
	≥University	242	12.5	
I find it disgusting and unacceptable (*n* = 1194; 54.2%)				
Sociodemographic variables	Category	*N*	(%)	*p*-Value
Sex	Male	334	44.1	0.000 ***
	Female	806	55.6	
Age (years)	18–34	493	50.0	0.015 *
	35–54	431	57.6	
	≥55	270	56.7	
Level of education	≤Secondary school	133	48.2	0.221
	≥University	1007	52.1	
I think it is forbidden and should not be consumed (not Halal) (*n* = 112; 5.1%)				
Sociodemographic variables	Category	*N*	(%)	*p*-Value
Sex	Male	65	8.6	0.000 ***
	Female	54	3.7	
Age (years)	18–34	58	5.8	0.047 *
	35–54	22	2.8	
	≥55	32	6.7	
Level of education	≤Secondary school	25	9.1	0.004 *
	≥University	94	4.9	
I am afraid of insects and cannot eat them (*n* = 228; 10.3%)				
Sociodemographic variables	Category	*N*	(%)	*p*-Value
Sex	Male	43	5.7	0.000 ***
	Female	231	15.9	
Age (years)	18–34	135	13.7	0.002 *
	35–54	64	8.5	
	≥55	29	6.0	
Level of education	≤Secondary school	26	9.4	0.167
	≥University	248	12.8	
I think there are better and healthier food options (*n* = 390; 17.6%)				
Sociodemographic variables	Category	*N*	(%)	*p*-Value
Sex	Male	176	23.2	0.000 ***
	Female	217	15.0	
Age (years)	18–34	177	17.9	0.947
	35–54	131	17.4	
	≥55	82	17.2	
Level of education	≤Secondary school	52	18.8	0.629
	≥University	341	17.7	

^a^ Chi-square test; * *p* < 0.05; *** *p* < 0.001.

As shown in Table 3, males were more willing (23%) compared to females (7.8%), who predominantly showed unwillingness (82.8%), a statistically significant difference (*p* = 0.000). Age also played a role, with younger individuals (18–34 years) demonstrating higher willingness (14.6%) than older groups with a significant association between age and willingness (*p* = 0.000). The education level showed a minimal influence on willingness, with similar patterns across the educational attainment levels meaning that there was no significant difference (*p* = 0.289).

Table 4 shows factors influencing EIs consumption based on socio-demographic characteristics. For food neophobia regarding EIs consumption as an alternative protein source, women achieved a significantly higher mean than men (*p* = 0.000). No significant difference was found between educational levels or age groups. The consumption of EIs as an alternative protein source and as an environmental concern was significantly higher among men than women (*p* = 0.000) and among younger age groups (18–34 years) (*p* = 0.000). Similar results were observed in the willingness to consume edible insects (EIs) as a sustainable food source, particularly when considering the health benefits associated with their consumption. The results showed that the level of positive beliefs about consuming EIs was significantly higher in men than in women (*p* = 0.000). There was also a significant difference in the level of positive beliefs regarding consuming EIs (*p* = 0.000) with those aged 18–34 years than in older age groups and individuals with a university education or higher (*p* = 0.016).

The multiple logistic regression analyses revealed key sociodemographic and attitudinal predictors for both willingness and uncertainty to consume EIs as an alternative protein source. The model demonstrated a strong overall fit, with a Nagelkerke R^2^ value of 0.731, indicating that 73.1% of the variance in willingness and uncertainty was explained by the included predictors. For willingness, being male (OR = 2.68, *p* = 0.000), younger (18–34 years, OR = 1.397, *p* = 0.004), and influenced by social effects (OR = 2.009, *p* = 0.000), environmental considerations (OR = 2.538, *p* = 0.000), health benefits (OR = 1.485, *p* = 0.000), and beliefs (OR = 1.579, *p* = 0.000) were significant predictors. For uncertainty, significant predictors included being male (OR = 1.754, *p* = 0.000), younger (18–34 years, OR = 1.037, *p* = 0.031), social effects (OR = 0.565, *p* = 0.017), barriers related to health risks (OR = 0.554, *p* = 0.025) and disgust (OR = 0.850, *p* = 0.031), environmental concerns (OR = 3.542, *p* = 0.000), health (OR = 1.109, *p* = 0.005), and beliefs (OR = 1.188, *p* = 0.000). Other attitudinal factors like familiarity, neophobia, and certain barriers did not show strong predictive power (Table 5).

## 4. Discussion

EIs provide a sustainable alternative protein source, are high in their amino acid profile, and have health and environmental benefits. Additionally, being eco-friendly with low carbon footprints makes them a sustainable approach for addressing global food security challenges. However, cultural barriers, familiarity, practices, and food neophobia continue to limit their acceptance.

Most Saudi participants (1912, 86.6%) reported not having any previous experience of consuming EIs or the opportunity to try EIs. Similar results have been reported from different European countries [35,36], where consumer hesitation may be owing to cultural factors and limited exposure to insect-based food products. Only 296 (13.4%) participants in this study reported having previous experience in consuming EIs, which was consistent with the 17.87% in a Slovenian consumer report [37]. This proportion is, however, less than that of previous studies conducted in Asian countries such as China (73.73%), Malaysia (35.6%), and Africa (97%) where participants have a long history of eating EIs as a cultural practice [24,38].

The level of acceptance of EIs is influenced by several demographic factors such as, sex, age, and educational level [35,36,37,38,39]; in the present study, men consistently showed higher acceptance rates for EIs consumption compared to women. Similarly, a study conducted among 537 Slovenian participants to evaluate the consumer perceptions and acceptance of EIs as an alternative protein source found higher acceptance among men (19.32%) compared to women (16.56%) [37]. In Hungary, similar results of acceptance occurred with significant differences between participants by sex (males vs. females, 7.2% vs. 2.9%), and acceptance was higher among the younger (18–39 years) age group [39]. In addition, younger Portuguese men accepted EIs consumption as an alternative protein source [40]. These results are consistent with our findings, indicating that men tend to try new foods more than women, are less neophobic, and are more curious and adventurous in tasting, as they tend to seek excitement.

The present study found greater resistance to accepting EIs as an alternative protein source among older participants, suggesting that younger participants are more receptive and have better EIs acceptance, as previously reported [41,42]. In contrast, a western Kenyan study found differences in the acceptance of EIs consumption, with it being higher among older participants than the younger groups [43]. This was attributed to younger groups being less familiar with EIs and less willing to consume them [42]. Furthermore, in Catalonia, Spain, a study that explored consumer opinions regarding EIs consumption revealed that participants aged 40–59 years were the most willing to consume EIs and incorporate them into their diets in the future [44].

The findings from the current study indicated no significant association between the education level and willingness to consume EIs, but this result should be interpreted with caution. The sample was highly skewed toward individuals with a university-level education (87.5%), limiting the educational variability and reducing the statistical power to detect meaningful differences. This homogeneity likely masked any potential relationship between education and an openness to novel food sources. The lack of influence of education observed in this study may also be attributed to the overriding role of cultural and religious norms in Saudi Arabia, regional differences in exposure to EIs, and the influence of stronger factors such as food neophobia and social norms. Furthermore, differences in the study design and context compared to previous research may explain the variation in the findings [39,42,45]. Prior studies have consistently shown that higher education correlates with greater environmental awareness, better nutritional knowledge, and reduced food neophobia, all factors that can support the acceptance of alternative proteins. Therefore, our findings do not necessarily contradict the existing literature, but rather reflect the limitations of our sample. Considering these limitations, the education level was excluded from the multiple regression analysis because it did not significantly contribute to the model’s explanatory power. Its inclusion did not improve the model fit or predictive accuracy due to the low variability within this variable. Future research with more diverse educational representation is needed to accurately assess the role of education in shaping attitudes toward EIs.

To indicate the familiarity of consuming EIs, participants were asked if they had ever met or heard of someone eating EIs; almost 80% of participants had encountered or heard of people eating EIs such as locusts. The willingness to consume EIs is strongly influenced by the cultural traditions of each country [46]. In countries with even a modest tradition of EIs consumption like Saudi Arabia, people are more open to incorporating EIs into their diets. Historically, crickets, locusts, and grasshoppers were commonly consumed in all Saudi Provinces and are recognized as a halal food that can be eaten as a traditional protein source to enhance nutrition [26].

Similarly, in a face-to-face interview study conducted in western Kenya that explored current knowledge regarding patterns of locust and grasshopper consumption and their potential to contribute significantly to human and animal nutrition, most participants were (51.6%) men, aged 18–40 years (64.0%), with formal education (95.9%). The participants, mostly from western Kenya (91.6%), were familiar with locusts as EIs and 51.2% had consumed EIs on a few occasions, although 48.2% had never consumed EIs. However, 35.8% were willing to consume EIs if the insect is mixed in foods such as bread and cakes. This finding indicates that the individuals from western Kenya have knowledge of edible locust species and that insect consumption is still a part of their culture; moreover, new participants are desirous to try novel foods [47].

The consumption of EIs is an important traditional practice in different regions of Africa, especially among communities familiar with the methods or techniques used in collecting and preparing the EIs as food. A greater proportion of respondents consuming EIs was reported in South Africa across villages in Limpopo (98%) and KwaZulu-Natal (64%) [48]. The older generation in rural areas traditionally passed on knowledge and information about EIs to the younger generation. The consumption of EIs has been declining owing to the adoption of Western diets and the availability of insects in the wild. Nevertheless, curiosity during childhood was often cited as a motivating factor for trying grasshoppers and other insects. Findings from previous studies indicate that communities with a history of EIs consumption and familiarity with their use are more receptive to including them in their diets. This cultural acceptance enhances the potential of EIs as a sustainable food source in the future.

Several barriers affect the consumption of EIs as alternative protein sources in the present study, including health concerns, psychological rejection, religious belief, fear of insects, and the availability of other food alternatives. A large proportion of participants (51.6%) expressed feelings of disgust and psychological rejection towards the idea of eating EIs, and the percentage was higher for those ≥ 55 years of age. These findings are consistent with that of a previous study which reported increased food disgust sensitivity with age [39,42]. This observation suggests that younger adults experience less disgust from food compared to older adults, as disgust is a powerful psychological factor that prevents participants from trying new, culturally unfamiliar foods. Additionally, our data indicated that participants with higher levels of food neophobia are more likely to experience disgust and perceive fewer general and specific risks and benefits associated with insect consumption than those with lower levels of food neophobia. Furthermore, a study conducted in Poland identified participants’ beliefs about EIs consumption and assessed the perception on food products containing added EI proteins to gain deeper insights into the role of food neophobia in the acceptance of insects as an alternative protein source. Food neophobia was higher in women (65.4%) than men (34.6%) and in those aged >60 years (57.4%) [30]. This is consistent with the 55.6% of women reporting disgust and rejection in our study compared to 44.1% of men. Also, rejection was higher (57.6%) in those aged 26–40 and >40 years (56.7%). This indicates a higher level of experience of food neophobia in women which increases with age.

Regarding the availability of healthier and better alternative protein sources, 17.8% indicated that they prefer traditional protein and believe better and healthier food options exist. In a multi-ethnic community in Singapore (10% Malay, 15% Indian, and 75% Chinese), the association between the sociodemographic factors and acceptance, attitudes towards the consumption of plant-based meat alternatives, cultured meat, and EI products was examined. The majority of participants (52%) were men who had a university education (52%) [49].

Respondents were more willing to consume plant-based meat alternatives and cultured meat than EI products, but the feeling of unnaturalness was the strongest barrier for acceptance [49]. Men were also more willing to consume the three types of alternative protein foods compared to women. Those with higher levels of education had a lower intention to consume alternative proteins in the form of insect-based products, which is consistent with our study. Additionally, 23.2% of men expressed that there are better and healthier food options compared to 15.0% of women [49]. However, our study did not show a significant difference (*p* < 0.629) between education levels and a preference for food alternatives.

Orkusz et al. conducted an online survey to study the level of fear of novel foods (EIs as an alternative source of protein) among 402 Polish students (35.2% men and 64.8% women) [50]. The study found that women (21.8%) demonstrated a higher level of fear compared to men (12.5%) toward novel foods. This finding is consistent with our study, where 15.9% of women expressed their fear towards eating EIs compared to 5.7% of men, indicating that fear may be the factor behind the lower acceptance of eating EIs among women.

On the contrary, different results were found in an online study conducted by Khatun et al. in Bangladesh involving 1014 participants, 68.4% men and 30.8% women, most of them (69.0%) being from the younger age group (18–30 years) [51]. Most of the participants (85.6%), regardless of their sex, age, and education level, were aware of the practice of eating EIs in different cultures and countries. More than half of the participants (68.0%) knew that EIs are a source of protein. Sex did not significantly influence food neophobia as the proportion of food neophobia was similar between men (40.3%) and women (41.7%). This result suggests that knowledge about EIs reduces the percentage of food neophobia in both sexes.

This study also showed that few participants (5.4%) consider eating EIs to be forbidden (not Halal); this belief was higher among men (8.6%) compared to women (3.7%) and those of the older age group (≥55 years). The belief that EIs are not Halal food comes from a lack of awareness about Islamic dietary rulings, misinformation about contamination or health risks, and regional variations in religious interpretations. In the modern Saudi society, where Western dietary habits are increasingly prevalent, traditional practices like consuming locusts are becoming less common. Men are more likely than women to perceive EIs as not Halal, possibly due to their roles as decision makers, greater engagement in religious discussions, and stronger adherence to traditional norms. To address these concerns, strategies should include educational campaigns on Islamic rulings, collaboration with religious scholars, culturally sensitive messaging, and gender-specific communication to promote EIs acceptance while respecting cultural and religious values

The consumption of EIs, particularly locusts, is deeply rooted in Arab history, as evidenced by historical narratives and cultural practices. In modern times, countries such as SA, Kuwait, Yemen, and Libya continue to regard the consumption of locusts as a traditional and customary practice [52]. Islam is the official religion in SA and Muslims are guided by dietary laws that dictate the consumption of “Halal” foods while avoiding “Haram” (forbidden) foods and beverages [52,53]. Locusts are recognized as a Halal food and the permissibility of consuming certain insects is further supported by the Hadith, where the Prophet Muhammad (peace be upon him) allowed the consumption of locusts. If the species of insect aligns with Islamic dietary principles, EIs present a viable, eco-friendly food source that aligns with the Islamic values of moderation and sustainability.

This observation explains why fewer participants (5.4%) consider eating EIs as forbidden. This belief was higher among men (8.6%) than women (3.7%) and was prevalent in older age groups, as in previous reports from other Muslim countries [49,52]. These results indicate that religious and cultural beliefs play a major role in the acceptance or rejection of the idea of eating insects as food. Endorsements and clarifications from respected religious scholars and fatwa authorities could significantly shape consumer attitudes and enhance public confidence in the permissibility of EIs. Clear guidance from trusted Islamic institutions or halal certification bodies can help dispel misconceptions and promote informed acceptance. Integrating religious messaging into future awareness campaigns may further align sustainability goals with cultural and spiritual values, fostering broader acceptance within Muslim communities.

Willingness to try EIs as an alternative protein source was considered a healthy and environmentally friendly option; this intention reflects openness to new sources of protein that may contribute to reducing environmental impacts. The findings indicate notable differences in willingness based on gender and age. Males were more willing compared to females, who largely exhibited unwillingness, highlighting a significant gender gap. Younger individuals (18–34 years) showed greater openness than older age groups, underscoring the importance of age as a factor. In addition, Australian participants who participated in an online survey measuring their willingness to eat EIs in the future reported a similar result to the present study, with 56.2% reporting their willingness to eat EIs in the future, with 73.4% being men and 50.9% women.

A Brazilian study identified a significant age-related trend, revealing that individuals aged 35–44 years demonstrated the highest willingness to consume edible insects (63.4%). Additionally, approximately 35.4% of participants had prior experience with insect consumption, with crickets and grasshoppers being the most commonly consumed varieties. Notably, participants expressed greater openness to trying edible insects when presented in alternative forms, such as chocolate-covered ants (52.1%) or insect-based flour (65.6%). These findings suggest that such innovative approaches could enhance the acceptance of insects as a sustainable protein source beyond initial expectations [28]. Previous experience in eating insects may lead to an increase in the consumer’s willingness to eat them in the future. Also, continuous exposure to the product in the market increases familiarity and desire.

Food neophobia in the present study was affected significantly by sex (*p* < 0.000), with women recording a higher mean score compared to men, and there were no significant differences in terms of the age groups or education level. Women’s higher food neophobia scores compared to men could be from psychological and social factors rather than age or education. Cultural expectations often encourage women to stick to traditional dietary practices, making them less open to unfamiliar foods like EIs. In contrast, men may be more willing to take risks with food choices due to cultural norms that promote adventurous behavior. Social pressures and fear of judgment can further discourage women from trying new foods, especially in environments where unfamiliar foods are viewed negatively. Addressing these barriers could involve strategies to reduce stigma and promote the cultural acceptance of novel foods.

In contrast, in Polish participants, food neophobia was affected significantly by sex (*p* < 0.001), age (*p* < 0.001), and education level (*p* < 0.026), highlighting notable differences in factors influencing food neophobia across cultures and communities [30].

In the present study, the environmental concern was influenced significantly by being male and in the younger age group. Furthermore, environmental concerns are not significantly affected by the education level. Men and younger participants showed greater environmental concern related to societal expectations and traditional roles that encourage them to engage with broader societal issues like sustainability. Cultural norms may also influence these differences, with men potentially viewing environmental responsibility as part of leadership. Understanding these dynamics could help develop better strategies to engage women and other groups in sustainability efforts and tailor environmental messages to resonate with different audiences.

Similar to the present study, Lebanese participants’ adoption of EIs as part of their diet was influenced significantly by their awareness of environmental sustainability, with a significant effect in the younger age and higher education level groups [54].

Health benefits are a major factor that influence the acceptance of EIs consumption as a rich source of protein and nutrients. Our study showed that health benefits were affected by sex and a younger age only. An online survey of 7222 participants from 14 countries examined the perceived nutritional benefits and health effects of edible insects, analyzing the influence of sociodemographic factors and living environments. The results revealed significant differences based on the gender (*p* < 0.001, with men showing higher interest), age (*p* < 0.001, particularly among individuals aged 18–30), and education level (*p* < 0.001, with greater interest among those holding university degrees). This finding may indicate that health and nutritional awareness is greater among educated participants, leading to their acceptance of EIs as a healthy food source [55]. However, in the present study, almost 90% of the participants held university degrees, which may explain the lack of significant effects on their perceptions of the health benefits of EIs consumption as a rich source of protein and nutrients.

Personal beliefs play an important role in participants’ acceptance of eating EIs, as they are affected by factors such as dietary habits, culture, and an awareness of their health benefits, in addition to the likelihood of trying them in the future. The results showed that the lower negative belief was affected by sex, with men recording a higher mean score than women. Likewise, a younger age and higher level of education were affected by a lower negative belief. In contrast to the present study, Guine et al. (2024) found that women (2.53 ± 0.76), older age groups (2.56 ± 0.77), and participants with a low-level education (2.56 ± 0.82) had more negative beliefs toward eating EIs [55]. These differences between the two studies suggest a difference in dietary habits and cultural and social distinctions between Saudi Arabians and the participants of this previous international study.

This study identified key factors influencing willingness and uncertainty toward consuming EIs among Saudi participants. The regression model explained about 73% of the variance in willingness. Males and younger adults (18–34 years) were more willing to accept EIs, driven by lower food neophobia and stronger beliefs in their health and environmental benefits. Positive attitudes like environmental concern, health awareness, and the social influence also increased acceptance. Uncertainty was linked to a younger age, being male, health concerns, disgust, and negative social perceptions, though environmental and health factors still had a positive impact. Familiarity with EIs and food neophobia has a limited influence, suggesting that cultural and contextual factors play a larger role. The findings highlight the importance of culturally tailored campaigns that focus on benefits while addressing social and psychological barriers to increase acceptance in Saudi society [29,49,56].

This present study has some limitations that may affect the generalizability of its results. First, a higher proportion of women may affect the balanced representation of opinions across the sexes. Women generally exhibit a greater interest in health, wellness, and nutrition, which increases their likelihood of participating in diet-related research, particularly studies focusing on eating behaviors. This pattern is especially noticeable in online survey-based research, where men are more likely to disengage or drop out early [57].

Second, data were collected through an electronic questionnaire using snowball sampling via social media, which facilitated rapid access to a large and diverse sample within a specific population, especially when traditional recruitment methods may be less effective or resource-intensive. However, this approach presents limitations that may affect the generalizability of the results, since participants may share similar sociodemographic or attitudinal characteristics. Third, the cross-sectional nature of the study design reflects the opinions of participants at a specific period without the ability to track potential changes in their attitudes toward insect consumption over the long term. Fourth, EIs consumption was more common in previous years, but is no longer part of the current dietary habits as Saudi consumers are increasingly adopting Western diets. Additionally, the absence of EI food products in the market may affect the participants’ opinions. The current study is reliant on self-reported, hypothetical scenarios, which may not accurately reflect real-world behaviors. Factors like taste, appearance, and the social context can influence actual consumption, creating a gap between stated intentions and real actions. Future research should use experimental methods, such as tasting trials or product exposure, to better assess genuine consumer responses when the products are available in the market.

Fifth, the snowball sampling method presents certain limitations, as it inherently relies on participants’ social networks. This reliance may introduce selection bias and restrict the diversity of the sample. For instance, individuals who are less active on social media or are part of less connected communities may be under-represented. To mitigate these limitations, we recruited participants from different age groups, genders, and socioeconomic statuses. Additionally, the large initial sample size (2229 respondents) provided a buffer to accommodate for potential biases and non-responses, ultimately resulting in the inclusion of 2208 participants after applying the eligibility criteria. Finally, the non-random nature of this sampling technique restricts the generalizability of the findings, as it may not fully capture the variability within the target population. To address these limitations, this study employed strategies to broaden the recruitment efforts, such as diversifying the initial participants and utilizing multiple social media platforms.

Despite these limitations, this study has several strengths; to the best of our knowledge, this is the first study to assess the acceptance of EIs as an alternative protein-rich food among participants from different regions of SA. This study included a large sample size of 2208 participants, enhancing the reliability of the results. This comprehensive approach provides valuable insights into the potential for EIs to be accepted as a sustainable food source within the diverse cultural and demographic area of SA. This study provides important data that can be used in developing future policies and strategies to enhance food security and sustainability.

## 5. Conclusions

This study demonstrates that demographic factors significantly influence Saudi participants’ willingness to accept EIs as alternative protein sources. Men and younger individuals were more receptive, demonstrating higher willingness driven by environmental awareness, health benefits, and curiosity toward novel foods. In contrast, women and older participants showed resistance due to psychological and cultural barriers such as food neophobia and perceptions of disgust. Addressing these barriers through targeted awareness campaigns that emphasize the nutritional, environmental, and sustainability benefits of EIs is essential. Furthermore, efforts to increase familiarity and foster positive experiences with EIs can boost willingness and facilitate the adoption of sustainable dietary habits in Saudi Arabia. To enhance this study’s applied value, future efforts should include practical implementation strategies aimed at overcoming these barriers. Educational campaigns tailored to specific demographic groups can help shift perceptions and promote acceptance. Additionally, innovative product development and culturally sensitive marketing approaches are necessary to accommodate diverse consumer preferences and address social norms. These actionable insights can guide policymakers and industry stakeholders in supporting the integration of edible insects into mainstream diets as a sustainable protein source. Future research should explore consumer preferences for EI-based products by conducting longitudinal studies to monitor attitude shifts over time and by including a broad range of demographic groups to gain a more complete insight into acceptance patterns.

## Figures and Tables

**Table 1:** Socio-demographic characteristics of the participants (*n* = 2208).

Variables	Total (*n* = 2208)	
Age, *n* (%)		
18–34	984	44.57
35–54	749	33.92
≥55	475	21.51
Sex		
Male, *n* (%)	758	34.3
Female, *n* (%)	1450	65.7
Levels of education		
≤Secondary school, *n* (%)	276	12.5
≥University, *n* (%)	1932	87.5
Employment status		
Unemployed, *n* (%)	454	20.6
Employed, *n* (%)	1754	79.4
Monthly income		
^a^ SR ≤ 10,000, *n* (%)	1870	84.7
SR > 10,000, *n* (%)	338	15.3
Region of residence		
Eastern, *n* (%)	701	31.7
Northern, *n* (%)	131	5.9
Western, *n* (%)	277	12.5
Southern, *n* (%)	373	16.9
Central, *n* (%)	726	32.9

^a^ Saudi Riyal.

**Table 3:** Associations between socio-demographic characteristics and willingness of acceptance of consuming EIs as alternative protein sources (*n* = 2208).

Sociodemographic Variables	Category	Willingness (*n*%)	Uncertainty (*n*%)	Unwillingness (*n*%)	*p*-Value
Sex	Male	174 (23%)	402 (53%)	182 (24%)	0.000 ***
	Female	113 (7.8%)	717 (49.8%)	620 (82.8%)	
Age (years)	18–34	144 (14.6%)	503 (51.1%)	337 (34.2%)	0.000 ***
	35–54	59 (7.4%)	379 (47.7%)	356 (44.9%)	
	≥55	36 (7.5%)	251 (53%)	188 (39.6%)	
Level of education	≤Secondary school	44 (15.9%)	137 (49.6%)	95 (34.4%)	0.289
	≥University	234 (12.6%)	982 (50.8%)	707 (36.6%)	

*** *p* < 0.001.

**Table 4:** Factors influencing edible insects consumption based on socio-demographic characteristics (*n* = 2208).

Sociodemographic Variables	Category	Food Neophobia	*p*-Value	Environment	*p*-Value	Willingness	*p*-Value	Health	*p*-Value	Belief	*p*-Value
		Mean ± SD		Mean ± SD		Mean ± SD		Mean ± SD		Mean ± SD	
^a^ Sex	Male	4.18 ± 0.98	0.000 ****	4.20 ± 2.13	0.000 ***	4.27 ± 3.80	0.000 ***	4.06 ± 2.38	0.000 ***	4.71 ± 2.25	0.000 ***
	Female	5.42 ± 1.15		3.26 ± 1.64		3.30 ± 2.85		3.67 ± 1.94		3.33 ± 1.78	
^b^ Age (years)	18–34	3.29 ± 1.04	0.077	3.69 ± 1.94	0.000 ***	3.41 ± 3.45	0.000 ***	3.87 ± 2.12	0.003 *	3.57 ± 1.98	0.000 ***
	35–54	5.19 ± 1.05		3.20 ± 1.55		3.27 ± 2.86		3.69 ± 2.04		3.14 ± 1.77	
	≥55	4.13 ± 1.10		3.31 ± 1.64		3.26 ± 2.87		3.26 ± 2.09		2.91 ± 2.11	
^a^ Level of education	≤Secondary school	4.26 ± 1.00	0.970	3.64 ± 1.91	0.626	3.01 ± 3.44	0.212	3.74 ± 2.14	0.605	3.50 ± 1.93	0.016 *
	≥University	4.25 ± 1.06		3.58 ± 1.87		2.94 ± 3.33		3.81 ± 2.10		3.19 ± 2.15	

^a^ *T*-test; ^b^ ANOVA test; * *p* < 0.05; *** *p* < 0.001, **** *p* < 0.0001.

**Table 5:** Predictors of acceptance of consuming EIs as an alternative protein source among participants (*n* = 2208).

Variables	Odds Ratio (OR)	95% Confidence Interval (CI)	*p*-Value
Willingness			
Sociodemographic factors			
Male	2.68	1.588–4.523	0.000 ***
18–34 years	1.397	0.407–4.795	0.004 *
35–54 years	1.21	0.304–4.815	0.787
Attitudinal factors			
Familiarity	0.998	0.508–1.958	0.994
Social Effects	2.009	0.110–3.981	0.000 ***
Barrier 1 (unsafe or health risks)	0.925	0.597–1.43	0.726
Barrier 2 (disgusting and unacceptable)	0.690	0.354–1.343	0.275
Barrier 3 (not Halal)	0.972	0.279–2.894	0.896
Barrier 4 (fear)	0.915	0.361–2.319	0.851
Food Neophobia	1.467	1.162–1.851	0.061
Environment	2.538	1.274–1.731	0.000 ***
Health	1.485	1.029–1.21	0.000 ***
Belief	1.579	1.343–1.865	0.000 ***
Uncertain ty			
Male	1.754	1.322–2.328	0.000 ***
18–34 years	1.037	0.630–1.709	0.031 *
35–54 years	0.775	0.443–1.355	0.371
Attitudinal factors			
Familiarity	1.027	0.772–1.367	0.853
Social Effects	0.565	0.353–0.903	0.017 *
Barrier 1 (unsafe or health risks)	0.554	0.331–0.928	0.025 *
Barrier 2 (disgusting and unacceptable)	0.850	0.585–1.235	0.031 *
Barrier 3 (not Halal)	0.855	0.455–1.706	0.626
Barrier 4 (fear)	0.784	0.485–1.268	0.321
Food Neophobia	1.109	0.979–1.257	0.104
Environment	3.542	2.564–4.730	0.000 ***
Health	1.109	1.032–1.191	0.005 *
Belief	1.188	1.084–1.302	0.000 ***

* *p* < 0.05; *** *p* < 0.001.

## Data Availability

The original contributions presented in the study are included in the article, further inquiries can be directed to the corresponding author.

## Abbreviations

The following abbreviations are used in this manuscript:

EIs	Edible insects
FAO	Food and Agriculture Organization
SDGs	Sustainable Development Goals
UN	United Nations
WHO	World Health Organization
